# Cost effectiveness of community-based therapeutic care for children with severe acute malnutrition in Zambia: decision tree model

**DOI:** 10.1186/1478-7547-7-2

**Published:** 2009-01-15

**Authors:** Max O Bachmann

**Affiliations:** 1 Medical School, University of East Anglia, Norwich, NR4 7TJ, UK

## Abstract

**Background:**

Children aged under five years with severe acute malnutrition (SAM) in Africa and Asia have high mortality rates without effective treatment. Primary care-based treatment of SAM can have good outcomes but its cost effectiveness is largely unknown.

**Method:**

This study estimated the cost effectiveness of community-based therapeutic care (CTC) for children with severe acute malnutrition in government primary health care centres in Lusaka, Zambia, compared to no care. A decision tree model compared the costs (in year 2008 international dollars) and outcomes of CTC to a hypothetical 'do-nothing' alternative. The primary outcomes were mortality within one year, and disability adjusted life years (DALYs) after surviving one year. Outcomes and health service costs of CTC were obtained from the CTC programme, local health services and World Health Organization (WHO) estimates of unit costs. Outcomes of doing nothing were estimated from published African cohort studies. Probabilistic and deterministic sensitivity analyses were done.

**Results:**

The mean cost of CTC per child was $203 (95% confidence interval (CI) $139–$274), of which ready to use therapeutic food (RUTF) cost 36%, health centre visits cost 13%, hospital admissions cost 17% and technical support while establishing the programme cost 34%. Expected death rates within one year of presentation were 9.2% with CTC and 20.8% with no treatment (risk difference 11.5% (95% CI 0.4–23.0%). CTC cost $1760 (95% CI $592–$10142) per life saved and $ 53 (95% CI $18–$306) per DALY gained. CTC was at least 80% likely to be cost effective if society was willing to pay at least $88 per DALY gained. Analyses were most sensitive to assumptions about mortality rates with no treatment, weeks of CTC per child and costs of purchasing RUTF.

**Conclusion:**

CTC is relatively cost effective compared to other priority health care interventions in developing countries, for a wide range of assumptions.

## Background

Children aged under five years with severe acute malnutrition (SAM) in Africa have high mortality rates without effective treatment [1-5]. Hospital inpatient treatment of SAM can reduce mortality [5], but in developing countries hospital treatment is too inaccessible and costly for most children with SAM. Community-based therapeutic care (CTC) is a recent model for early diagnosis and treatment of SAM in ambulatory primary health care settings. The key nutritional component of SAM treatment is ready to use therapeutic food (RUTF). This is a nutrient-dense food with a nutrient content/100 kcal that is similar to F100 milk, the diet recommended by the World Health Organization (WHO) in the recovery phase of the SAM treatment [6]. A major advantage of RUTF over F100 is that it contains little water and is thus resistant to microbial contamination, and suitable for storage and use at home without refrigeration [7]. Other key components of CTC are simplified clinical protocols, decentralised provision, community mobilisation and high population coverage [8]. CTC also includes supplementary feeding for moderate malnutrition, which is not considered in this study [8]

Several home-based RUTF programmes in developing countries have shown good outcomes [8-10]. However there have been few controlled trials comparing mortality rates with other treatments [11,12]. No trials have prospectively compared CTC with no treatment, which would be unethical. Although resources constraints are critical for the expansion of CTC, we are aware of only one published study reporting original data on costs of ambulatory treatment of severe acute malnutrition in a developing country [13]. That trial, with 437 children in Bangladesh in 1990 and 1991, showed that inpatient care cost $156 per child, day care cost $59 and domiciliary care cost $29.

Lusaka, Zambia, provides an innovative example of large scale provision of CTC through government primary health care centres. Since 2005 the Lusaka District Health Management Team (LDHMT), which is responsible for the city's 25 primary health care centres, has steadily expanded CTC provision by its staff working in these health centres. By January 2008, 21 of the 25 LDHMT health centres were providing CTC. CTC clinics were set up within each health centre, each staffed by a nurse, a health educator and two volunteers. Nurses were trained to diagnose SAM in children under 5 years of age, by measuring mid upper arm circumference (MUAC) and examining children for bilateral pedal pitting oedema. SAM was defined as MUAC of 11 cm or less, or bilateral pitting oedema [8]. Children were treated with RUTF of 200 kcal/Kg/day, broad-spectrum antibiotics, vitamin A, folic acid, anti-helminthics and, if indicated, anti-malarial treatment [8]. They were then asked to return weekly until they had recovered. Recovery was defined as having MUAC > 11 cm, weight gain and no oedema for at least two weeks, and clinically well [8]. Children with initial MUAC of 11 cm or less at admission were supposed to receive at least 8 weeks treatment, although in practice duration of treatment varied. Children were referred to hospital for inpatient care if they failed to respond to treatment, deteriorated or were severely ill and required hospital care.

Twenty volunteers attached to each health centre screened children at the health centres and in the community and referred those with SAM to CTC. Government and private sector nurses and traditional health practitioners working near to each health centre were trained and encouraged to identify and refer SAM cases. Popular art theatre discussions were held to raise community awareness of SAM and CTC. Valid International, a company specialising in nutritional research in developing countries, helped initiate the programme and provided technical support to the LDHMT for implementation and staff training. RUTF was manufactured in Lusaka or imported, and delivered to LDHMT medical stores free of charge.

The aims of this study were 1) to describe the outcomes of CTC in Lusaka, 2) to estimate the costs of CTC and 3) to estimate the effectiveness and cost effectiveness of this type of CTC, compared to no treatment. The reasons for comparing CTC to no treatment were, first, to enable the cost-effectiveness of CTC to be compared to any other health care intervention and, second, because comparable data on costs of alternative ways of treating SAM in this population were not available.

## Methods

The study was a cost effectiveness analysis based on a decision tree model [14]. Cost and cost effectiveness were considered from the perspective of health services. The existing model of care was compared to a hypothetical alternative of providing no treatment [15]. Household and societal costs of illness and care were beyond the scope of this study.

### Decision tree

**Figure 1:**
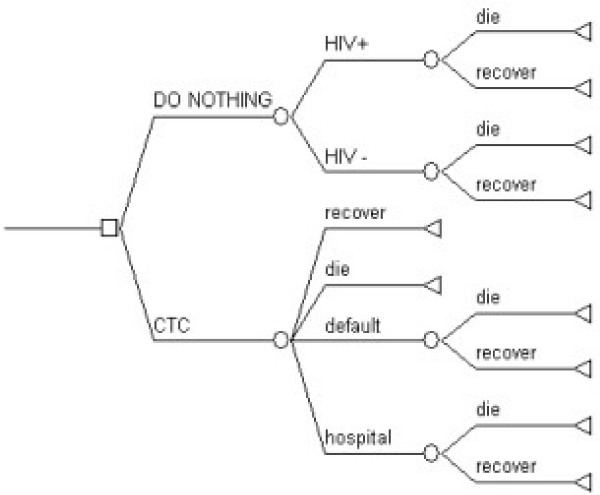
**Decision tree**.

The structure of the decision tree is shown in Figure 1. The square represents choice, circles represent chance (probabilities) and triangles represent outcomes. There are two options: "do nothing" or "CTC". For each option, various things could happen to each child, leading ultimately to recovery or death. For the "do nothing" option, death rates differed according to whether children were HIV infected or not. For the "CTC" option, HIV status was not considered, because the effects of HIV/AIDS were already incorporated into known CTC outcomes, and because the HIV status of most children receiving CTC was not known. Children receiving CTC in health centres could have one of the 4 outcomes known to the CTC programme. Children referred to hospital, and children who defaulted, then either died or recovered. For each option, the probability of each outcome was entered into the model to calculate expected rates of death or recovery. For the CTC option, costs of CTC and of hospital treatment were also entered into the model. The "do nothing" option was assumed to cost health services nothing. Effectiveness of CTC was calculated as the difference in death rates between the two options. The cost of CTC, divided by the effectiveness of CTC, is the incremental cost effectiveness ratio, expressed in dollars per life saved. Assuming that each child who recovers has a life expectancy of 33.3 disability adjusted life years [5], cost effectiveness was also expressed in dollars per DALY gained.

### Model parameters: probabilities of outcomes

Model parameters are shown in Table 1. The primary outcomes of interest were mortality up to year after developing SAM, and expected DALYs after surviving one year.

**Table 1:** Assumptions, distributions and sources of model parameters

Parameter	Mean	Standard error*	Source and comments
Outcomes			
*Do nothing option*			
Mortality without CTC (HIV-)	0.18	0.045	[1-4] SE assumed.
Relative risk of death with HIV, no CTC	2.0	0.5	[18] SE assumed
Prevalence of HIV in under fives	0.15	0.0375	[16,17] SE assumed.
*CTC option*			
Death rate during CTC	0.026	0.0032	Programme data.
Proportion defaulting from CTC	0.172	0.0075	Programme data.
Death rate in defaulters from CTC	0.058	0.029	Assumed. SE set so 95% CI is +/- 100% of mean
Hospital referral rate from CTC	0.059	0.0047	Programme data.
Death rate in hospital	0.37	0.093	UTH data. SE assumed
Mortality within a year of recovery	0.0364	0.0091	[19]
Expected DALYs if child recovers	33.3	NA	[5]
			
Costs (CTC option only)			
No. weeks of CTC – recovered	6.6	1.6	Programme data.
No. weeks of CTC – referred	4.8	1.1	Programme data.
No. weeks of CTC – died	3.6	1.6	Programme data.
No. weeks of CTC – defaulted	5.1	1.5	Programme data.
Cost per health centre visit	$4.24	$1.06	LDMHT. SE assumed.
Cost per kg of RUTF	$6.10	$1.53	Valid International. SE assumed.
Kg of RUTF per week per child	1.90	0.016	Programme data.
Cost of community mobilisation per child	$1.06	$0.27	LDMHT. SE assumed
Valid cost per child	$68.69	$17.17	Valid International. SE assumed.
Cost per day in hospital	$41.35	$10.34	[24] SE assumed.
Days in hospital	14	3.5	UTH data. SE assumed.

* SE standard errors for probabilistic sensitivity analysis; normal distributions assumed.

CI confidence interval. $ international dollars, year 2008. NA not applicable. UTH Lusaka University Teaching Hospital

For the 'do nothing' option, expected mortality was based on evidence from a review of child mortality rates associated with malnutrition in developing countries [1]. In particular we based mortality rates on rigorous community based cohort studies conducted in Malawi [1,2] and Uganda [3,4] in the late 1980s that used MUAC as a predictor of mortality. In the Lusaka CTC population, the median MUAC in children with MUACs of 11 cm or less was 10.6 (interquartile range 10.0–10.8) cm and in children with oedema the median was 12.0 (interquartile range 11.2–13.0) cm. Mortality rates with bilateral pedal oedema were assumed to be the same as with MUAC of 11 cm or less, as we found in this CTC programme. At the time of the cohort studies [1-4], for such children minimal treatment was available and the prevalence of HIV was negligible. Non-CTC children were therefore stratified by HIV status, to account for the increased death rate with HIV, the prevalence of which has increased over the past 20 years. The HIV prevalence estimates was based on numbers of infected children in Zambia, from UNAIDS [16] and numbers of children aged under 5 from the Zambia census [17]. Mortality with HIV was assumed to be double that without HIV [18].

For the CTC option, outcomes at the end of health centre care were death, recovery, referral to hospital, or default (Table 1). These outcomes were known from programme data for 2523 patients treated from September 2005 to September 2007. Among children referred from CTC to hospital, the death rate was assumed to be 37%, which was the death rate in the University Teaching Hospital (UHT) acute malnutrition ward (personal communication Dr B Amadi, UHT paediatrician). The death rate among children who defaulted from CTC was assumed to be the same as for all other children, including those referred to hospital, because they had similar prognostic characteristics. That is, among children who defaulted, mean initial MUAC was 11.2 cm and 25.2% had oedema; among children who did not default, mean initial MUAC was 11.4 cm and 25.3% had oedema. The cohort estimates of mortality used for the "do nothing" option were based on one year of follow up, but CTC programme data were based on an average of 7 weeks of follow-up. To be able to compare annual mortality rates between the two options we therefore assumed that CTC patients who did not die during CTC or in hospital had the same annual mortality rate as all children aged under 5 in Zambia. The under five mortality rate in Zambia in 2006 was 182 per thousand live births [19]. Therefore we assumed that 3.64% (0.182/5) of children who recovered during CTC would die within a year.

### Model parameters: cost of CTC and hospital care

All costs were expressed in international dollars for the year 2008. Unit costs measured in Zambian kwacha and UK pounds were deflated to their year 2000 equivalents [20,21], then converted to year 2000 international dollars using WHO exchange rates to reflect purchasing power parity [22]. They were then adjusted to year 2008 values using United States inflation rates from 2000 to 2008 [23].

The relevant types of cost were for health centre visits, RUTF, hospital admissions and Valid International's contribution to establishing the programme. Costs per health centre visit were based on the 2008 LDHMT budget, minus the proportion of the LDHMT budget devoted to non-health centre services (and the proportional administration costs), plus LDHMT staff salaries paid by the provincial health department. This total annual cost of health centre care was then divided by the number of health centre visits during 2007 to produce an average cost per health centre visit. The cost of community mobilisation was estimated from the 2008 LDHMT budget for community based child health activities, 10% of which was assumed to be for CTC (personal communication, Dr C Mbwili, LDHMT). This was multiplied by 2.4 years of the CTC programme and divided by the 3358 children treated, producing a mean cost of community mobilisation per child. The cost per kilogram of RUTF in Zambia was estimated by Valid International. Programme data showed that the mean body weight per child was 7.4 (standard error 0.065) Kg, and that each child received 200 kcal/Kg/day, which is equivalent to 1.9 (standard error 0.016) Kg RUTF per week. Costs of ambulatory CTC were health centre unit costs plus RUTF costs, multiplied by the duration of treatment. Treatment duration was stratified by CTC outcome (Table 1).

Valid International expenditure on the Zambia programme was reported from April 2005 to January 2008. This excluded RUTF production (which was already accounted for), and included administration, training, research, local and international travel, and consultancy. For each line item of expenditure, the proportion attributable to CTC was estimated by two senior Valid International personnel. This was divided by the number of children who received CTC over the same period, to produce an average cost to Valid International per child, regardless of duration of treatment.

Costs per day of hospital inpatient care at the Lusaka University Teaching Hospital were not available and so were based on WHO estimates of tertiary hospital care in Zambia, adjusted to include drug costs [24]. For children referred to hospital, these daily costs were multiplied by the average length of stay in the Lusaka University Teaching Hospital acute admission ward (personal communication, Dr B Amadi).

### Analysis

The cost effectiveness analysis was carried out with Tree Age Pro Healthcare software and checked with Microsoft Excel. Point estimates were calculated for costs, outcomes, CTC effect (that is, difference in mortality) and incremental cost effectiveness ratios (CTC costs divided by CTC effect), using the point estimates for each model parameter.

Probabilistic sensitivity analyses [14,25] were conducted with Tree Age Pro Healthcare, to quantify the combined uncertainty about costs, effects, and cost effectiveness, based on the uncertainty about all of the model's parameters. First, the distribution of each parameter was defined (Table 1). Parameter standard errors were assumed to have normal distributions and were estimated from programme data where available. If not available, to be consistent standard errors were defined as 25% of the mean, so that 95% confidence intervals would be 50% more or less than the mean; the only exception was for mortality among defaulters, for which the standard error was larger to reflect greater uncertainty. Monte Carlo simulation was performed, with 10000 iterations per analysis. From simulation results we estimated 95% confidence intervals for each output of the model from their percentiles. We also calculated the probability that the intervention was cost effective for a range of values that society might be willing to pay to obtain one unit of effect (that is, dollars per life saved or per DALY gained).

Finally, one- and two-way sensitivity analyses were conducted. One way sensitivity analyses were calculated using the mean values of each parameter, and varying the values of one parameter at a time. Two way sensitivity analyses were conducted to examine the effects of simultaneously varying the values of two parameters.

Patient's consent and research ethics committee approval were not necessary because the study was based on aggregate programme data and published literature and did not require access to individual patient records.

## Results

CTC cost an average of $203 per child, 70% of which was due to RUTF and Valid International's costs (Tables 2 and 3). Health centre visits and hospital admissions accounted for 30% of the CTC costs. Of Valid International's costs, 51% were for personnel, 42% were for travel and subsistence, and 7% were for other items.

**Table 2:** Mean costs of community-based therapeutic care per child

Cost item	Unit cost ($)	Mean number of items per child	Mean cost per child ($)	% of total
RUTF (Kg)	6.20	11.70	72.52	35.8

Technical support	68.69	1.00	68.69	33.9

Hospital per day	41.35	0.83	34.16	16.9

Health centre visits	4.24	6.16	26.10	12.9

Community mobilisation	0.66	1.0	1.06	0.5

Total			202.53	100.0

RUTF ready to use therapeutic food

**Table 3:** Costs and effects of community-based therapeutic care compared to no treatment

	CTC		No treatment		Difference	
	Mean	(95% CI)	Mean	(95% CI)	Mean	(95% CI)

Mean cost per patient ($)	203	(139–274)	0	0	203	(139–274)

Death rate (%)	9.2	(4.3–7.25)	20.8	(10.5–31.8)	11.5	(0.4–23.0)

Expected DALYs*	30.2	(29.3–31.2)	26.4	(22.7–29.8)	3.8	(0.14–7.7)

CTC community-based therapeutic care.

* assuming 33.3 disability adjusted life years (DALYs) expected after surviving one year [5]

**Figure 2:**
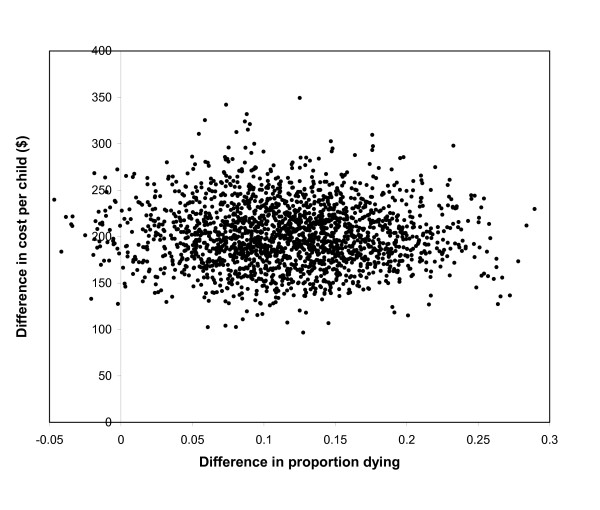
**Incremental costs and effects from Monte Carlo simulation**.

The results of the Monte Carlo simulation, from which confidence intervals and probabilities of cost effectiveness were estimate, are shown in Figure 2. Each dot represents the cost and effect of CTC for each iteration. The expected mortality rates after one year were 9.2% with CTC and 20.7% with no treatment – a risk difference of 11.5% (Table 3). Thus one life would be saved for every 8.7 children who received CTC. The average increase in expected DALYs with CTC was 3.8 per child. The relative risk of death with CTC compared to doing nothing was 0.44 (95% CI 0.26–0.95).

The cost of CTC was $1760 (95% CI $592–$10142) per life saved and $ 53 (95% CI $18–$306) per DALY gained.

**Figure 3:**
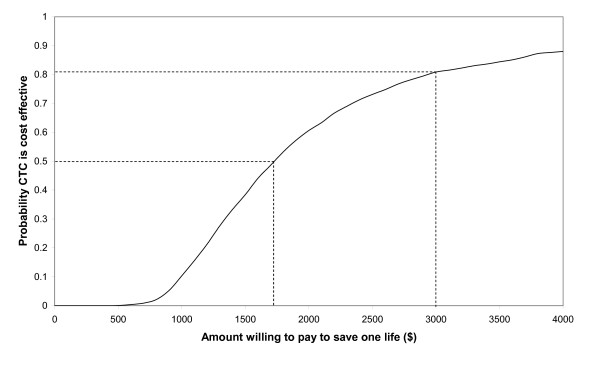
**Probability CTC was cost effective for different amounts willing to pay per life saved**.

**Figure 4:**
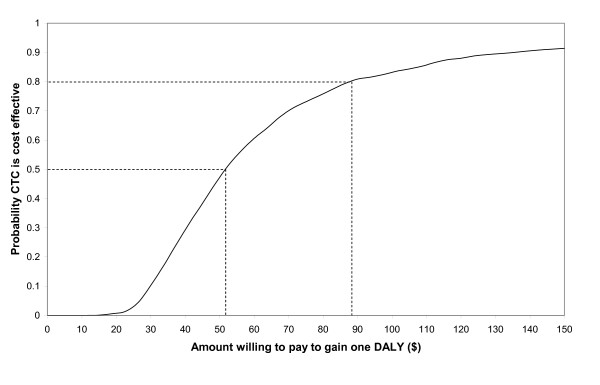
**Probability CTC was cost effective for different amounts willing to pay per DALY gained**.

CTC was more likely than not to be cost effective if society was willing to pay at least $1700 per life year gained (Figure 3). CTC was more than 80% likely to be cost effective if society was willing to pay at least $3000 per life saved. With regard to DALYs, CTC was more likely than not to be cost effective if society was willing to pay at least $52 per DALY gained (Figure 4). CTC was more than 80% likely to be cost effective if society was willing to pay at least $88 per DALY gained.

**Figure 5:**
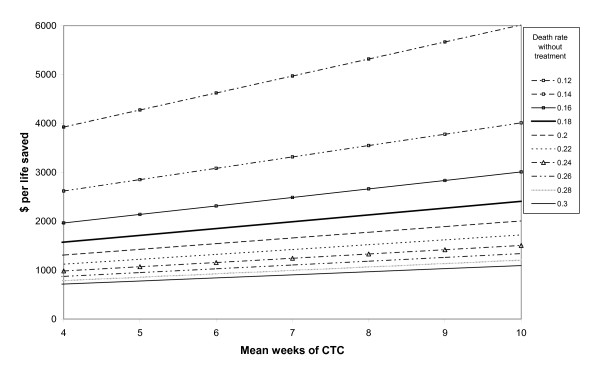
**Cost per life saved for different assumptions about death rates without treatment and number of weeks of CTC per child**.

**Figure 6:**
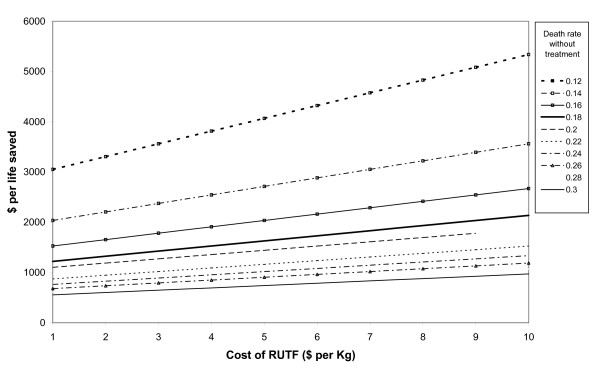
**Cost per life saved for different assumptions about death rates without treatment and costs per kilogram of RUTF**.

The model was most sensitive to assumptions about expected mortality without treatment, weeks of CTC per child, effect of HIV on mortality without CTC, hospital referral rate, cost per kilogram of RUTF, quantity of RUTF consumed per week and technical support costs (Table 4). Cost effectiveness estimates were less sensitive to assumed unit costs of health centre visits and hospital admissions. The CTC outcome parameter which was least well known – death rates among defaulters – had relatively little influence on cost effectiveness estimates. The model's sensitivity to combinations of the most influential variables is shown in Figures 5 and 6. They show that the cost per life saved increased exponentially as the assumed death rate without treatment decreased towards 12%, and increased linearly with increasing weeks of CTC per child and cost per kilogram of RUTF.

**Table 4:** Sensitivity analysis: cost per life saved for different values of model parameters

	**Parameter values**			**Incremental cost effectiveness ratio ($ per life saved)**		
	**Base**	**Minus 50%**	**Plus 50%**	**Low**	**High**	**Range**
**Outcome probabilities**						
Death rate within a year without CTC HIV- *	0.18	0.09**	0.27	17502**	927	16576
Relative risk of death if HIV+, no CTC *	2	1	3	2300	1426	874
Refer to hospital from CTC	0.059	0.030	0.089	1479	2100	622
Death within a year if recover with CTC	0.0364	0.0182	0.0546	1520	2091	571
HIV prevalence*	0.15	0.075	0.225	1994	1575	419
Death during CTC	0.026	0.013	0.039	1586	1978	392
Death rate in hospital	0.37	0.185	0.555	1608	1944	337
Death rate among defaulters	0.045	0.022	0.067	1703	1821	118
Default from CTC	0.173	0.087	0.260	1721	1802	82
**Costs**						
Weeks of CTC – Recovered	6.6	3.3	9.9	1419	2101	682
RUTF per Kg ($)	6.20	3.10	9.30	1445	2075	630
Mean Kg of RUTF per child per week	1.90	0.95	2.85	1445	2075	630
Valid cost per child ($)	68.69	34	103	1462	2058	597
Hospital cost per day ($)	41.35	20.68	62.03	1612	1908	297
Days in hospital	14	7	21	1612	1908	297
Costs per health centre visit ($)	4.24	2.12	6.36	1647	1873	227
Weeks of CTC – Defaulted	5.1	2.6	7.7	1699	1821	123
Weeks of CTC – Referred	4.8	2.4	7.2	1740	1780	39
Weeks of CTC – Died	3.6	1.8	5.4	1754	1767	13
Community mobilisation per child $	1.06	0.5	1.6	1755	1765	9

* CTC is more cost effective if these parameters are greater.

** note higher mortality with CTC than with no care

## Discussion

The study shows that CTC for SAM among children aged under five years in Lusaka results in good outcomes at a reasonable cost. The estimated cost of $1760 per life saved, or $53 per DALY gained, suggests that this model of CTC has a similar cost effectiveness to other priority child health interventions in Africa such as immunisation, micronutrient supplementation, and treatment of pneumonia and diarrhoea [26]. The cost per DALY gained was very similar to the result of a World Bank study based in Guinea in 1998 [27], despite the different methods of evaluation. WHO has classified child health interventions as highly cost effective if the cost per DALY gained is less than the country's gross national product per capita [15,26]. This can be used as one indicator of society's willingness (or at least ability) to pay for improved health. This supports CTC, since Zambia's gross national income per person per year was $1000 in 2006 [28].

The main strengths of this study are that 1) it was based on an innovative large scale programme implemented through government primary care health centres throughout a Zambian city, 2) it had original and up to date programme data on costs, outcomes and severity of SAM and 3) it compared the costs and outcomes of the programme with what would be expected without any intervention. Comparison with no treatment allows the cost effectiveness of this model of CTC to be compared with any other intervention in health, and not just to be considered as an incremental change to alternative nutrition strategies [15,29]. This method of comparing a health care intervention with the hypothetical alternative of doing nothing, and using probabilistic sensitivity analyses, follows WHO health economists' recommendations for economic evaluation and priority setting [15,30].

The main limitation of comparing CTC to doing nothing is that it is dependent on assumptions about the outcomes and costs of no health care for SAM. We do not know what these children's mortality rates would have been without treatment. Most recent studies of mortality among children with SAM have been among children who received treatment [5,9,10]. We therefore relied on population based cohort studies in Africa carried out about 20 years ago when little health and nutritional care was available [1-4]. However, we have accounted for the likely effect of the HIV epidemic, and UNICEF estimates indicate that under five mortality rates in Zambia remained constant between 1990 and 2006 [31]. Although comparable mortality rates were not available for children with oedema, this programme's data showed that mortality among children with oedema was the same as for children with MUAC of 11 cm or less and with no oedema. Our assumption of 18% mortality without treatment does not seem excessive considering that, in 9 randomised trials of hospital treatment of SAM, short term mortality rates ranged from 16% – 46% (median 20%) among control group children who received conventional treatment [5]. Recent African hospital case series have also shown higher short term mortality rates for children with MUAC < 11.5 cm, despite treatment [18,32,33]. Furthermore, UNICEF estimates [31] show that under five mortality rates in Zambia were higher than in Malawi and Uganda where the cohort studies were conducted. Even if we assumed that mortality rates without treatment were as low as 12%, CTC was still relatively cost effective, with a cost per life saved of about $5000 (Figure 5), and with a cost per DALY gained of $150.

The deterministic sensitivity analyses (Table 4) showed that results were most sensitive to assumptions about mortality without treatment (discussed above), costs of RUTF, and costs of technical support, which has implications for future research priorities. The importance of mortality without CTC highlights the need for future studies to track mortality in comparator populations. RUTF accounted for 36% of total CTC costs, underlining the desirability of reducing RUTF costs in future. It seems likely that an alternative less costly RUTF formulation, using locally grown soya, sorghum and maize instead of imported milk powder and peanuts, could be as effective at lower cost. Larger scale production and food procurement would also be likely to reduce costs in future. This alternative will be evaluated in a randomised trial and economic evaluation soon to be started in this setting. The relatively high cost per child of Valid International's input was largely due to technical support while setting up the programme. At least some of these costs could thus have been considered as capital costs and spread over a longer period, as the programme becomes increasingly run by government health services. In future Valid International's emphasis will change from ongoing technical support to training health ministry trainers, which could be less costly. Thus costing of longer term and larger scale implementation will be needed in future.

Several other limitations and assumptions of the study should be considered. First, it would have been desirable also to have compared CTC with alternative ways of treating SAM. As stated in the Background, although other studies have compared outcomes of community- and hospital-based treatment of SAM [11,12], only one study compared costs [13]. If community based care was at least as effective as hospital-based care [13], it is plausible that the former would be less costly and thus more cost effective. However we could not make such a comparison with local data because our examination of clinic records found that comparable outcome measures prior to CTC were not available. Second, unit costs of hospital and health centre care were not available specifically for children with SAM. However neither of these unit costs had much influence on overall cost and cost effectiveness estimates (Table 4). Third, the mortality rate among children who defaulted was unknown but this too had relatively little influence. Fourth, HIV prevalence among this population was not known. If HIV prevalence was higher than the 15% assumed, that would increase the estimated cost effectiveness of CTC by increasing the mortality rate without CTC. Fifth, DALY estimates assumed that health and life expectancy would return to normal after recovery, although it is plausible that children who recovered would be at higher risk of stunting and poorer health in future [5]. Alternatively, they could be relatively hardy survivors. However, it is easy for readers to adjust these effectiveness and cost effectiveness estimates to reflect different assumptions about expected DALYs after recovery. For example, if one assumed that 25 DALYs were expected after recovery, instead of 33.3, the cost per DALY gained would be $53 × 33.3/25 = $71. Finally, discounting was not used because child level costs covered less a year and expected DALYS were assumed to be net present values.

## Conclusion

The Lusaka model of CTC for SAM appears highly cost effective. This study suggests that this form of CTC should be expanded to the rest of Zambia and adapted for other African countries with high rates of SAM. Cost effectiveness could be increased in future with less external technical support, as CTC is increasingly implemented through government services, and by reducing RUTF costs through local and larger scale production and sourcing of components. Priorities for future research include controlled trials and economic evaluations of alternative ways of providing CTC, such as hospital-based care or selective SAM-only programmes. This requires prospective collection of individual level data on severity of SAM, HIV status, use of health services and outcomes, and active follow up of children who default.

## Abbreviations

CTC: community-based therapeutic care; LDHMT: Lusaka District Health Management Team; MUAC: mid upper arm circumference; RUTF: ready to use therapeutic food; SAM: severe acute malnutrition; UNICEF: United Nations Children's Fund; WHO: Word Health Organization,

## Competing interests

The author received funding from Valid International and Concern to carry out the study.

## Authors' contributions

MOB designed the model, identified relevant parameters and data sources, carried out the analyses and wrote the paper.

## Authors' information

Max Bachmann is a health services researcher and public health physician who uses health economic and clinical epidemiological methods to evaluate innovative health care interventions to improve population health, such as child health care, HIV/AIDS care and chronic disease management, in Africa and the United Kingdom. Now at the University of East Anglia, he previously worked at the medical schools of the universities of Cape Town, Bristol and the Free State.

## References

[B1] Pelletier DL (1994). The relationship between child anthropometry and mortality in developing countries: implications for policy programs and future research. J Nutr.

[B2] Pelletier DL, Low JW, Johnson C, Msukwa LA (1994). Child anthropometry and mortality in Malawi: testing for effect modification by age and length of follow-up and confounding by socioeconomic factors. J Nutr.

[B3] Vella V, Tomkins A, Ndiku J, Marshal T, Cortinovis I (1994). Anthropometry as a predictor for mortality among Ugandan children, allowing for socio-economic variables. Eur J Clin Nutr.

[B4] Vella V, Tomkins A, Borghesi A, Migliori GB, Ndiku J, Adriko BC (1993). Anthropometry and childhood mortality in northwest and southwest Uganda. Am J Publ Health.

[B5] Bhutta Z, Ahmed T, Black RE, Cousens S, Dewey K, Giugliani E, Haider BA, Kirkwood B, Morris SS, Sachdev HPS, Shekar M, Maternal and Child Undernutrition Study Group (2008). What works? Interventions for maternal and child undernutrition and survival. Lancet.

[B6] Briend A, Lacsala R, Prudhon C, Mounier B, Grellety Y, Golden MH (1999). Ready-to-use therapeutic food for treatment of marasmus. Lancet.

[B7] Briend A (2001). Highly nutrient-dense spreads: a new approach to delivering multiple micronutrients to high-risk groups. Br J Nutr.

[B8] Bahwere P, Binns P, Collins S, Dent N, Guerrero S, Hallam A, Khara T, Lee J, Mollison S, Myatt M, Saboyo M, Sadler K, Walsh A (2006). Community Based Therapeutic Care A Field Manual.

[B9] Collins S, Dent N, Binns P, Bahwere P, Sadler K, Hallam A (2006). Management of severe acute malnutrition in children. Lancet.

[B10] Ashworth A (2006). Efficacy and effectiveness of community-based treatment of severe malnutrition. Food Nutr Bull.

[B11] Ciliberto MA, Sandige H, Ndekha MJ, Ndekha MJ, Ashorn P, Briend A, Ciliberto HM, Manary MJ (2005). Comparison of home-based therapy with ready-to-use therapy with standard therapy in the treatment of malnourished Malawian children: a controlled, health centre effectiveness trial. Am J Clin Nutr.

[B12] Patel MP, Sandige HL, Ndekha MJ, Briend A, Ashorn P, Manary MJ (2005). Supplemental feeding with ready-to-use therapeutic food in Malawian children at risk of malnutrition. J Health Pop Nutr.

[B13] Ashworth A, Khanum S (1997). Cost-effective treatment for severely malnourished children: what it the best approach?. Health Policy Plan.

[B14] Drummond MF, Sculpher MJ, Torrance GW, O'Brien B, Stoddart GL (2005). Methods for the Economic Evaluation of Health Care Programmes.

[B15] Tan-Torres Edejer T, Baltussen R, Adam T, Hutubessy R, Acharya A, Evans DB, Murray CJL, (Eds) (2003). Making Choices in Health: WHO Guide To Cost-Effectiveness Analysis.

[B16] UNAIDS/WHO Global HIV/AIDS Online Database. 2006 Report on the Global AIDS Epidemic. http://www.unaids.org.

[B17] Central Statistics Office Zambia. Census Data from Zambia. http://www.zamstats.gov.zm/census.php.

[B18] Amadi B, Kelly P, Mwiya M, Mulwazi E, Sianongo S, Changwe F, Thomson M, Hachungula J, Watuka A, Walker-Smith J, Chintu C (2001). Intestinal and systemic infection, HIV, and mortality in Zambian children with persistent diarrhoea and malnutrition. J Ped Gastr Nutr.

[B19] UNICEF (2008). State of the World's Children 2008.

[B20] Bank of Zambia. Snapshot Inflation. http://www.boz.zm/snapshot_inflation.htm.

[B21] Bank of England. Inflation Calculator. http://www.bankofengland.co.uk/education/inflation/calculator/flash/index.htm.

[B22] WHO-CHOICE. CHOosing Interventions that are Cost Effective. http://www.who.int/choice/costs/en/.

[B23] U.S. Department of Labor. Bureau of Labor Statistics. Consumer Price Index. http://www.bls.gov/CPI/.

[B24] Adam T, Evans DB, Murray CJ (2003). Econometric estimation of country-specific hospital costs. Cost Effect Res Allocation.

[B25] Ades AE, Claxton K, Sculpher M (2006). Evidence synthesis, parameter correlation and probabilistic sensitivity analysis. Health Econs.

[B26] Tan-Torres Edejer T, Aikins M, Black R, Wolfson L, Hutubessy R, Evans DB (2005). Cost effectiveness analysis of strategies for child health in developing countries. BMJ.

[B27] Prabhat JHA, Bangohoura O, Ranson K (1998). The cost-effectiveness of forty health interventions in Guinea. Health Policy Plan.

[B28] World Bank (2008). World Development Report 2008.

[B29] Hutubessy R, Chisholm D, Tan-Torres Edejer T, WHO-CHOICE (2003). Generalized cost-effectiveness analysis for national-level priority setting in the health sector. Cost Effect Res Allocation.

[B30] Baltussen RMPM, Hutubessy RCW, Evans DB, Murray CLJ (2002). Uncertainty in cost-effectiveness analysis: probabilistic uncertainty analysis and stochastic league tables. Int J Technol Assess Health.

[B31] UNICEF (2007). The State of the World's Children 2008 Child Survival.

[B32] Berkley J, Mwangi I, Griffiths K, Ahmed I, Mithwani S, English M, Newton C, Maitland K (2005). Assessment of severe malnutrition among hospitalized children in rural Kenya: comparison of weight for height and mid upper arm circumference. JAMA.

[B33] Bitwe R, Dramaix M, Hennart P (2006). Simplified prognostic model of overall intrahospital mortality of children in central Africa. Trop Med Int Health.

